# Sabotage Detection Using DL Models on EEG Data From a Cognitive-Motor Integration Task

**DOI:** 10.3389/fnhum.2021.662875

**Published:** 2021-10-08

**Authors:** Mahima Chaudhary, Meaghan S. Adams, Sumona Mukhopadhyay, Marin Litoiu, Lauren E. Sergio

**Affiliations:** ^1^Lassonde School of Engineering, York University, Toronto, ON, Canada; ^2^Faculty of Health, York University, Toronto, ON, Canada; ^3^KITE - Toronto Rehabilitation Institute, University Health Network, Toronto, ON, Canada

**Keywords:** deep learning, CNN, LSTM, wearable devices, baseline testing, cognitive-motor integration

## Abstract

Objective clinical tools, including cognitive-motor integration (CMI) tasks, have the potential to improve concussion rehabilitation by helping to determine whether or not a concussion has occurred. In order to be useful, however, an individual must put forth their best effort. In this study, we have proposed a novel method to detect the difference in cortical activity between best effort (no-sabotage) and willful under-performance (sabotage) using a deep learning (DL) approach on the electroencephalogram (EEG) signals. The EEG signals from a wearable four-channel headband were acquired during a CMI task. Each participant completed sabotage and no-sabotage conditions in random order. A multi-channel convolutional neural network with long short term memory (CNN-LSTM) model with self-attention has been used to perform the time-series classification into sabotage and no-sabotage, by transforming the time-series into two-dimensional (2D) image-based scalogram representations. This approach allows the inspection of frequency-based, and temporal features of EEG, and the use of a multi-channel model facilitates in capturing correlation and causality between different EEG channels. By treating the 2D scalogram as an image, we show that the trained CNN-LSTM classifier based on automated visual analysis can achieve high levels of discrimination and an overall accuracy of 98.71% in case of intra-subject classification, as well as low false-positive rates. The average intra-subject accuracy obtained was 92.8%, and the average inter-subject accuracy was 86.15%. These results indicate that our proposed model performed well on the data of all subjects. We also compare the scalogram-based results with the results that we obtained by using raw time-series, showing that scalogram-based gave better performance. Our method can be applied in clinical applications such as baseline testing, assessing the current state of injury and recovery tracking and industrial applications like monitoring performance deterioration in workplaces.

## 1. Introduction

Mild traumatic brain injuries (mTBI) or concussions have become an increasing public health concern, affecting an estimated 42 million individuals annually (Gardner and Yaffe, 2015). As a brain injury, concussion affects many aspects of function, including sensory, motor, and cognitive domains, and can thus have major implications for participation in activities of daily living. Approximately 15–20% of those who sustain concussions develop chronic symptoms and functional impairments that persist for months or years (Ellis et al., 2014; McCrory et al., 2017). As a result, individuals affected by concussion are seeking clinical and rehabilitation care in greater numbers than ever, highlighting the need for such care to reflect evidence based on objective metrics.

One issue with the current state of injury assessment and recovery tracking is its reliance on self-report. By their nature, symptoms are subjective: There is no way to measure a headache other than to record what an individual reports. However, there is evidence that subjective symptoms may resolve before full neurological recovery has occurred, leading to vulnerability to further injury (Hurtubise et al., 2016; Sergio et al., 2020). Deficits have been reported in a wide range of laboratory and neurophysiological outcome measures in asymptomatic individuals after concussion (Thériault et al., 2009; Slobounov et al., 2012; Baker and Cinelli, 2014; Tapper et al., 2016; Adams et al., 2020; Manning et al., 2020). This reliance of self-reported symptoms, therefore, means that diagnosis is less precise, intervention targets are more difficult to identify, and determining recovery is less clear than would be possible with more objective measures.

A second issue around assessment and recovery tracking at present is the use of pre-injury assessments. Objective measures applied to the same person pre-and post-injury would be a useful metric to assess injury effects and monitor recovery, since they would allow for more personalized care. However, because of their unreliability, the utility of “baseline measures” often collected annually for those in athletics or lines of duty has been called into question (Higgins et al., 2018; Rebchuk et al., 2020). A primary contributor to this concern is the potential for individuals to sabotage or willfully under perform their baseline tests (i.e., deliberately perform poorly to erode or incapacitate the assessment). For a variety of personal or social factors, such as a desire to return to work, duty, sport, or the pressures of impending litigation, individuals may not perform to the best of their ability. Thus, any injury/recovery assessment approach using baseline measures is only effective if both the pre- and post-injury assessments capture the best effort of an individual. To this end, a way to detect and prevent sabotage during baseline testing would add considerable reliability to concussion assessment and recovery metrics, improving care and preventing further injury.

Electroencephalography (EEG) has been used in several previous studies examining deceit detection. There are many studies that have worked on the problem of lie detection using EEG data obtained while participants completed the Guilty Knowledge Test (GKT), a psychophysiological questioning technique that can be used to determine whether a person is lying especially during a polygraph test. Abootalebi et al. (2008) acquired data from participants while they answered GKT questions, extracted various morphological and frequency-based features from the EEG tracing, and then selectively fed features to linear discriminant analysis algorithm for classification. They achieved 86% correct detection of total subjects. Deep belief network (Hinton et al., 2006) was used for deceit classification by Bablani et al. (2018) based on GKT, and the highest subject accuracy achieved was 83.4% using 16 channel EEG. A new machine learning method referred to as F-score-based extreme learning machine (ELM) was proposed by Gao et al. (2013) to classify lying and truth-telling. The methodology used a nine-channel EEG system to obtain signals during a GKT-based task and achieved best accuracy of 98.97%. A support vector machine (SVM) model was used to detect lies by Simbolon et al. (2015) during a stimulus display task, achieving a best accuracy of 70.83% in this study. Cakmak and Zeki (2015) classified the two states, i.e., lie and deception based on the EEG tracings, while the participants were shown Pokemon cards and asked whether or not a card belonged to them. They used short-time fourier transform and multi-layer perceptron for classification and achieved the accuracy of around 90%.

However, no studies have examined changes in the time-frequency scalograms of EEG signals in response to sabotaging a cognitive-motor integration task (a form of visuomotor skill assessment). In our approach, we propose to use scalograms of the EEG data as the input to our model. Previous studies examining sabotage detection have all used traditional EEG systems, collecting data from a minimum of nine scalp electrodes. An important focus when considering the application of our work to clinical concussion care and to workplace/clinic assessment is the use of technologies that are deployable in clinical environments. To this end, we used a portable EEG headband (Muse2™, InteraXon Inc., Toronto, Canada), a commercially available and consumer-grade device, to collect our EEG data. To our knowledge, this is the first time a portable EEG system has been used to detect sabotage. Here, we use a deep learning (DL) (Ian et al., 2016) approach to analyze EEG spectral data. DL is a subfield of machine learning (ML) (Michie, 1968), which is in turn a form of artificial intelligence (NJ, 2014) that focuses on teaching computers how to learn without the need to be programmed for specific tasks by training them on some examples or data. DL is a class of machine learning algorithms that uses complex multi-layered neural networks for a variety of tasks, where the level of abstraction increases gradually by non-linear transformations of input data. We have employed a multi-channel attention-based CNN-LSTM (Hochreiter and Schmidhuber., 1997; Krizhevsky et al., 2012) model to identify sabotage, and the model has been trained on EEG data from a cognitive-motor integration (CMI) task. A variant of the proposed model has been used in the past (Ordóñez and Roggen, 2016; Kim et al., 2019) for classifying time-series data. Similar multi-channel models have been lately used to classify time-series data (Ruffini et al., 2019; Mukhopadhyay and Banerjee, 2020; Mukhopadhyay and Litoiu, 2020). However, unlike models used in the past, our model uses multi-channel CNN that accepts inputs from all the EEG channels at the same time and also applies self-attention (Vaswani et al., 2017) to the data that helps our model to identify and focus on the data that is relatively more important for the given problem.

The objective of this study was to determine the possibility of distinguishing willful underperformance (“sabotage”) from best effort (“no-sabotage”) using a DL analysis of neurophysiological data collected during a visuomotor skill assessment. We hypothesized that these EEG spectral measures of neural activity during the intentionally poor performance on a CMI task would be significantly different from a maximal effort performance. The rest of the study is organized as follows: section 2 shows our methods. The evaluation metrics used in our study are discussed in section 3. The results achieved are shown in Section 4. Finally, section 5 presents the discussion and future work.

## 2. Methods

In our proposed methodology, we first collected data from participants performing a CMI task. We then performed data cleaning, data preprocessing, and feature engineering on data. The transformed data were fed to the model. We explain each step in detail.

### 2.1. Participants

Electroencephalogram and behavioral data were collected from 12 healthy volunteers (8 female and 4 male) aged 22–50 years of age. Participants had no history of substance abuse, neurological illness or impairment, brain injury, psychoactive drug treatment, or concussion. All procedures were approved by the York University's Human Participants Research Committee, and all participants provided informed consent to participate.

### 2.2. Experimental Task

**Figure 1:**
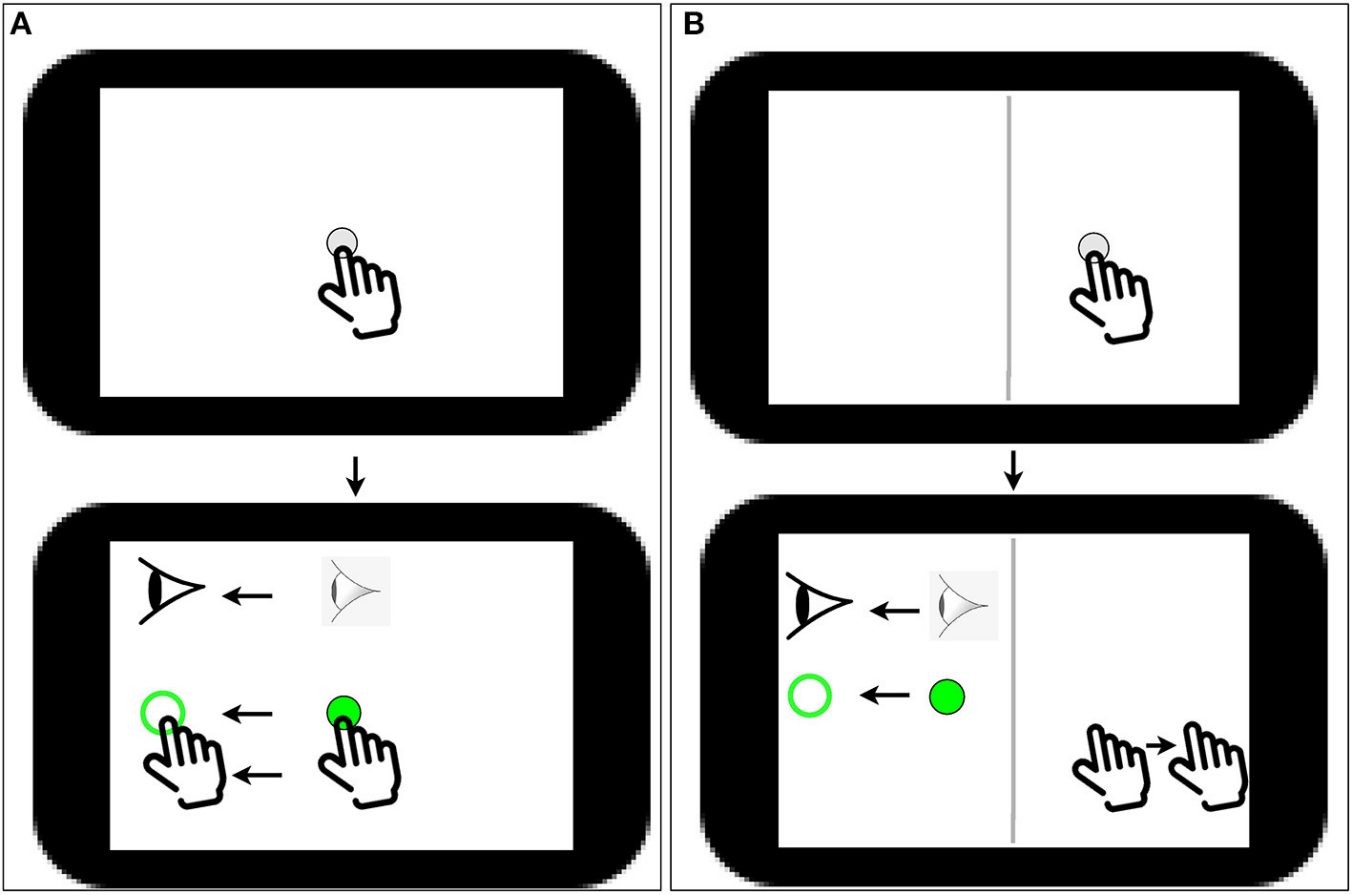
**(A)** Task 1 (simple sliding finger along the same direction); a white dot of 5 mm diameter appears on screen followed by a solid green 8 mm diameter circle in the center of the screen; the user has to then slide the solid green dot to an open green peripheral target. **(B)** Task 2, A CMI task: A vertical white line (shown as dark gray in the image) divides the screen in to two halves; a white dot of 5 mm diameter appears on the right half of the screen followed by a solid green on the left side of the screen; the user has to then slide the solid green dot to an open green peripheral target by sliding the finger in the opposite direction of the open green target on the right side of the screen.

Participants completed two blocks of a computer-based visuomotor skill assessment task (BrDI™) that included one standard and one non-standard conditions where vision and action were decoupled. This latter condition required the integration of spatial and cognitive rules and thus required cognitive-motor integration (CMI). Participants sat at a desk so they could comfortably reach a 10.1 inch tablet (Samsung Galaxy Tab A) placed on the desk in front of them. All hand movements were made on the tablet. The task required participants move the index finger of their dominant hand along the touch screen of the tablet to move a cursor (white dot, 5 mm diameter) from a central location to one of four peripheral targets (up, down, left, or right relative to center) as quickly and as accurately as possible. To start a trial, participants guided the cursor to a solid green 8 mm diameter circle in the center of the screen. After a 2,000-ms center hold time, an open green peripheral 10 mm diameter target was presented, which served as the “Go” signal for the participant to initiate movement. The participant slid their finger along the touch screen to move the solid green cursor onto the open green target. Once the cursor reached and remained in the peripheral target for 500 ms, it disappeared, signaling the end of the trial. The next trial began with the presentation of the central target after an intertrial interval of 2,000 ms. Peripheral targets on the tablet were located 37.5 mm from the central start target (center-to-center distance). There were 20 trials for each task, 5 to each target. In the standard condition, participants looked at the target and used their finger to displace the cursor that was directly under their finger, thereby directly interacting with the targets. In the non-standard CMI condition, the display was split by a vertical white line. The participant had to view the targets, and the cursor presented in the left half of the tablet screen. To displace the cursor in this task, however, they had to slide their finger within the right blank half of the tablet screen. Furthermore, the cursor feedback was 180° rotated from finger motion, such that the participant had to slide one direction to move the cursor in the opposite direction to reach the target. They were instructed to view the targets on the upper screen and not their (extrafoveally located) hand in the blank lower screen. Participants completed two practice trials in each of the four directions before each task was presented for the first time in order to become familiar with the task requirements. The task performed by the participants is shown in Figure 1.

For each condition, they were instructed to either complete the task as quickly and as accurately as possible (true effort condition) or to willfully perform poorly while still completing the trials (sabotage condition). In summary, participants completed two sets (true effort condition, and sabotage condition) of the two tasks (standard task, and CMI task), for a total of 80 trials. The entire behavioral task took approximately 10 min (2–3 min for each 20-trial individual task). Both the conditions and the standard vs. CMI task within a condition were randomized for each participant.

### 2.3. Data Acquisition and Recording

**Figure 2:**
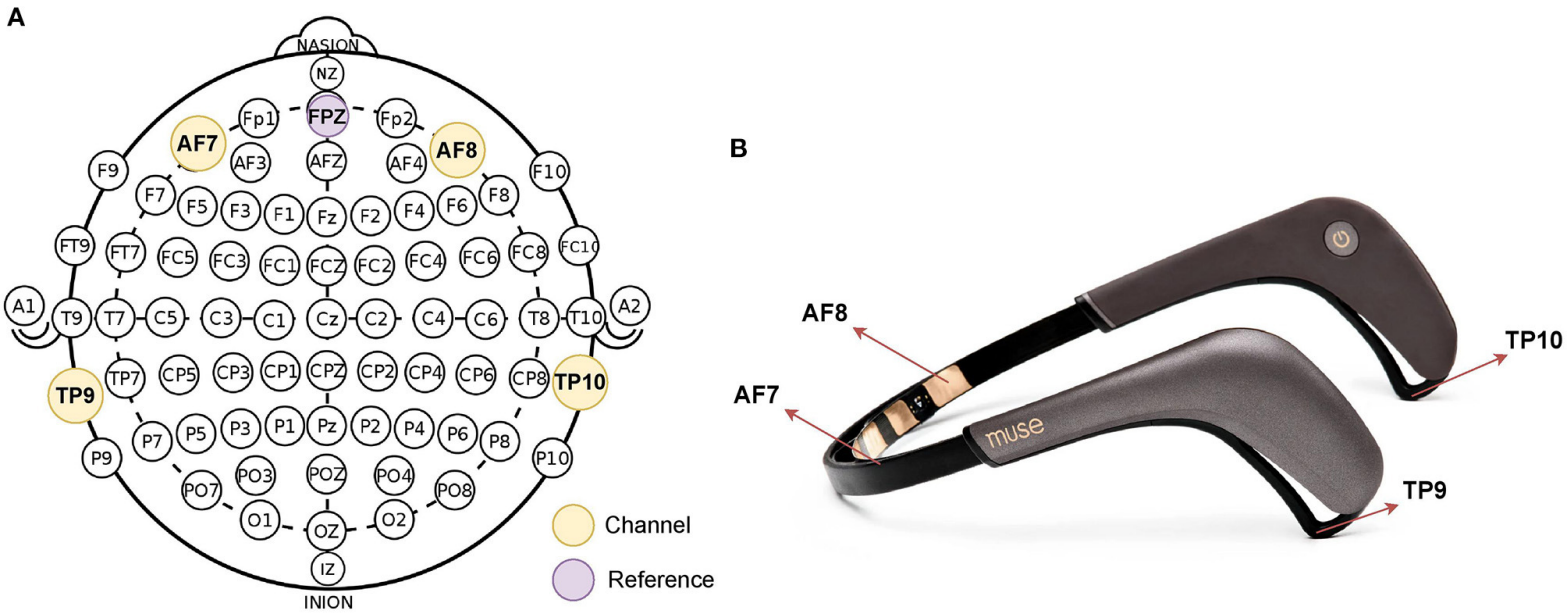
**(A)** The 10-20 system of electrode placement for Muse. **(B)** Muse 2 Headband.

During the completion of the CMI task, EEG data were collected from a portable EEG headband system (Muse2™, InteraXon Inc., Toronto, Canada) using Mind Monitor software (mind-monitor.com) using Open Sound Control Protocol (Freed and Schmeder, 2009). The Muse2 device recorded continuous EEG data from four electrodes: TP9, TP10, AF7, and AF8, which are placed in accordance with the International 10–20 System for electrode placement. Figure 2A shows 10–20 system 2 placement for Muse, and Figure 2B shows Muse 2 headband. The EEG data from Muse 2 are collected at a frequency of 256 Hz. Data were collected and saved for subsequent analysis. The “marker” function was used on the Mind Monitor software to denote the start of the behavioral task. The recording software was manually stopped immediately following completion of the last trial.

### 2.4. Data Cleaning and Preprocessing

Artifacts such as blinks, muscle contractions, or eye movements were labeled by Matlab's EEGLAB software (Delorme and Makeig, 2004) and Mind Monitor application. They were then removed manually. We also divided the data into small time windows of 50 ms and computed the variance of data in each window. If it was more than a selected threshold (which was equal to plus or minus 2 SD of the mean), the time window was flagged and later discarded. We also examined the data of the individual subject and used the trial that had a minimum number of jaw clenches and eye blinks (which were detected using Mind Monitor) for further experimentation. We made sure that there was proper contact with the electrodes with the help of Mind Monitor application as it lets you know if you have not made sufficient contact with the skin on the contact points. For better conductivity, we also applied some water to the electrodes before the start of the experiment. The data from the four channels were normalized before extracting features in time-frequency domain.

### 2.5. Feature Extraction

**Figure 3:**
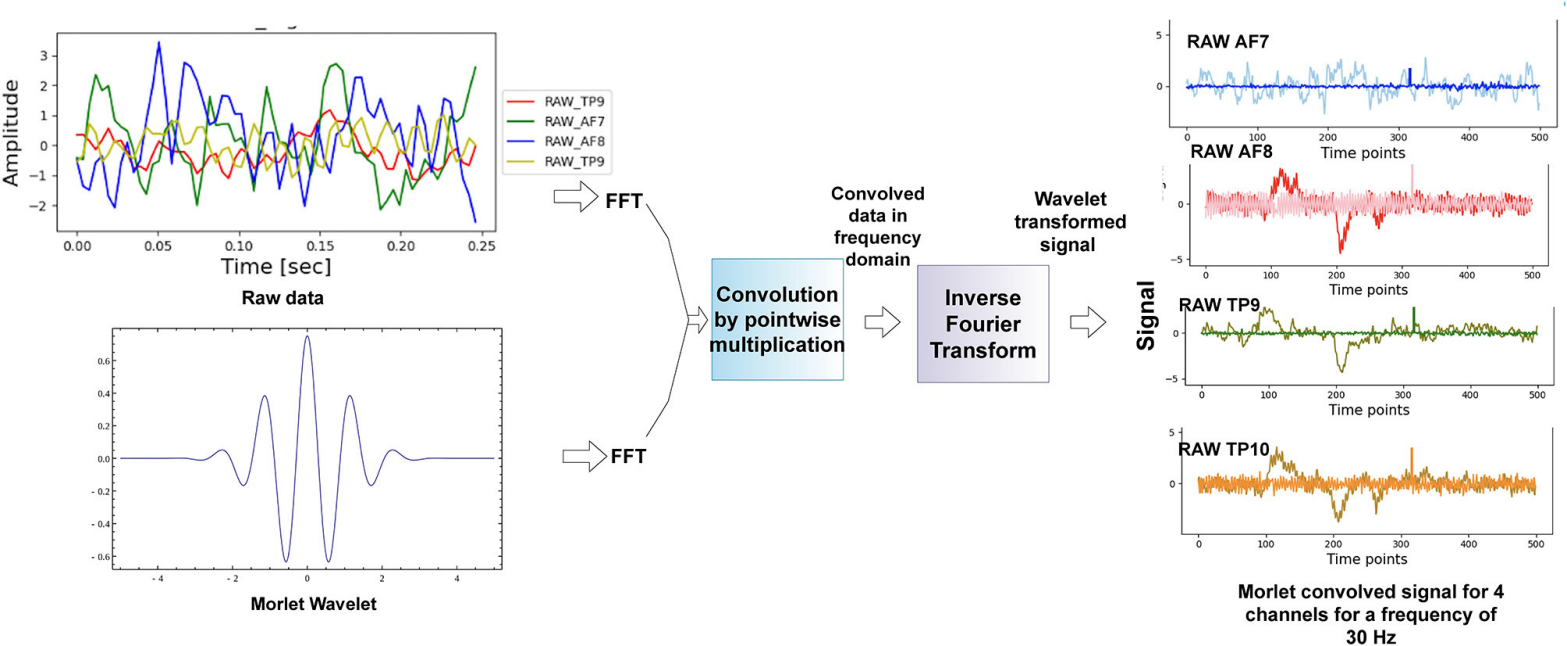
Methodology used for scalogram conversion. The technique of fast fourier transform was used to get the time-frequency representation of the data.

**Figure 4:**
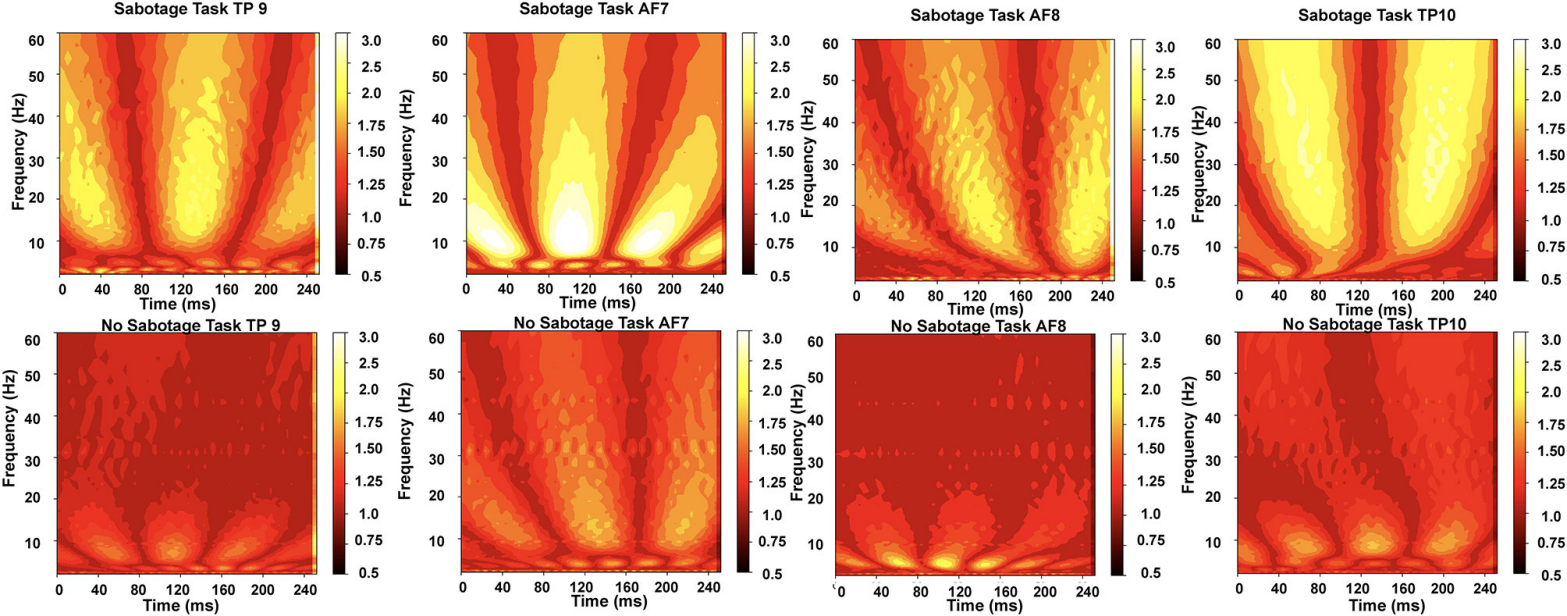
The normalized scalograms obtained for the two tasks from the four channels; we can observe that higher frequencies (alpha to beta measured in Hz) are more active for sabotage task than for no-sabotage task.

We have applied continuous wavelet transform as done in Wang et al. (2010), Hyeon and Choi (2019), and Chaudhary et al. (2020) using a morlet wavelet on the raw data from the four channels of the Muse 2 headband. Figure 3 shows the approach we used to get the morlet convolved signal from which we generated scalograms, and Figure 4 has a visualization of the normalized scalograms. A Morlet wavelet was obtained by the multiplication of a Gaussian with a sine wave and is described by the following equation.


(1) $$\begin{array}{l} {\text{Ψ}}_{\sigma }\left(t\right)=c_{\sigma }\pi^{-\frac{1}{4}}e^{-\frac{1}{2}t^{2}}\left(e^{i\sigma t}-\kappa_{\sigma }\right) \end{array}$$

where $\kappa_{\sigma }=e^{-\frac{1}{2}\sigma^{2}}$, σ is duration of the wavelet, and the normalization constant *c*_σ_ is:


(2) $$\begin{array}{l} c_{\sigma }={\left(1+e^{-\sigma^{2}}-2e^{-\frac{3}{4}\sigma^{2}}\right)}^{-\frac{1}{2}} \end{array}$$

The Morlet wavelet helped to reduce edge artifacts and noise from the data. It also helped to obtain a balance in temporal precision and frequency precision. The technique of Fast fourier transform (FFT) (Nussbaumer, 1982) has been used to convert the data into time-frequency domain. We first performed FFT on the raw data to convert it into frequency domain as shown in Equation 3.


(3) $$\begin{array}{l} \hat{x}\left(\omega \right)=\int_{-\infty }^{+\infty }x\left(t\right)e^{-i\omega t}dt \end{array}$$

where *x*(*t*) is the time series signal, and then, we performed FFT on the Morlet wavelet (the wavelet was formed from the frequency of interest) following Equation 4.


(4) $$\begin{array}{l} {\hat{\text{Ψ}}}_{\sigma }\left(\omega \right)=c_{\sigma }\pi^{-\frac{1}{4}}\left(e^{-\frac{1}{2}{\left(\sigma -\omega \right)}^{2}}-\kappa_{\sigma }e^{-\frac{1}{2}\omega^{2}}\right) \end{array}$$

After the two signals were obtained in the frequency domain, we performed point-wise multiplication to get the entire signal in frequency domain. The data were then converted back to time domain using inverse Fourier transform using Equation 5.


(5) $$\begin{array}{l} \text{Cw=}\frac{1}{2\pi }\int_{-\infty }^{+\infty }\hat{x}\left(\omega \right){\hat{\text{Ψ}}}_{\sigma }\left(\omega \right)d\omega \end{array}$$

**Figure 5:**
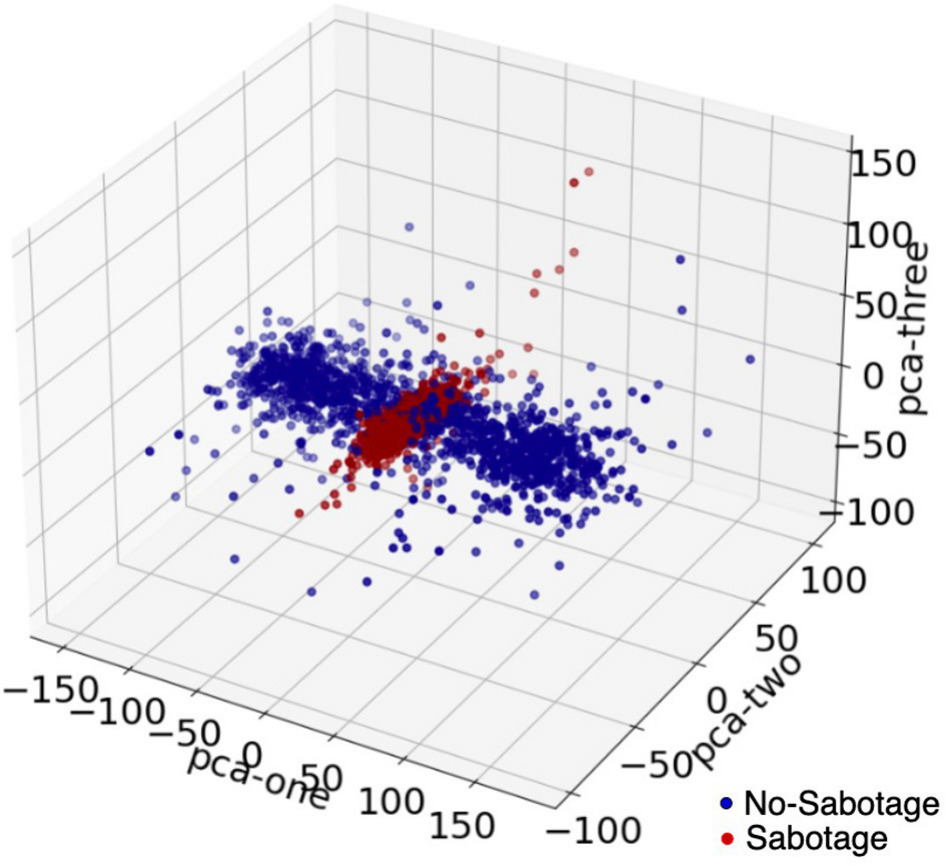
The visualization of data using PCA in 3D; the above data are for Subject 1.

The power of this signal was then calculated by finding the magnitude of the complex signal and then squaring it to get the absolute power component of the signal. The data were then divided into overlapping (50% overlap) windows, each consisting of 64 time points. A window size of 64 samples was chosen after experimenting with different window sizes. An overlap of 50% was taken for each window, and this was also chosen after experimenting with no-overlap and different overlap sizes. We discuss this in detail in the results section. We made scalograms of dimension 64 by 64 from this time-frequency transformation. For each scalogram representation of the data, we firstly considered the data for a time window of 0.25 s (64 timepoints) and then we took data with 50% overlap, thus next window was from 0.125 to 0.375 and then 0.25 to 0.50 and so on. The window sizes were kept constant throughout. We did this for all the four channels, and we gave all the four scalograms as input to our model. In Figure 5, we see principle component analysis (PCA) visualization of the data into two classes, such as sabotage and no-sabotage, which are in well-defined clusters.

### 2.6. Deep Learning Approach

**Figure 6:**
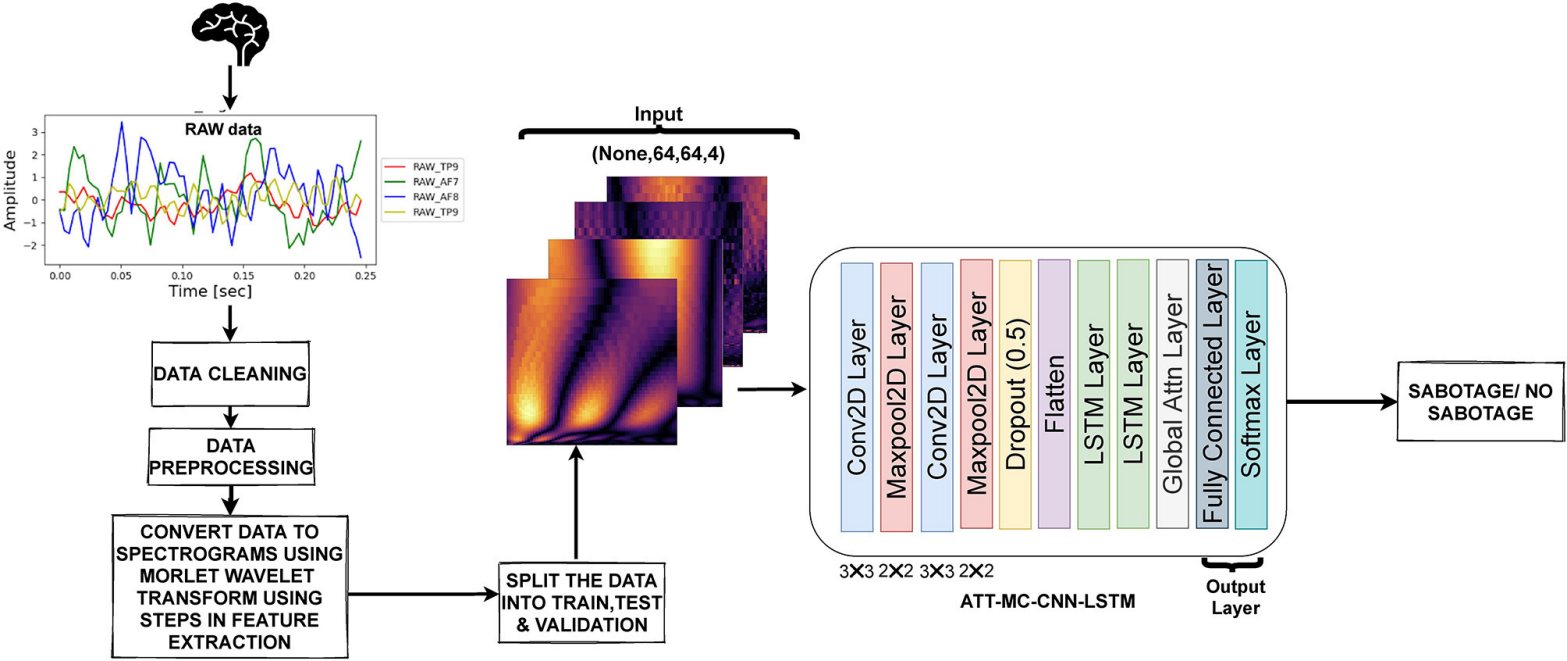
The methodology used in our approach. The raw data obtained were preprocessed, and scalograms were generated from the data. The scalograms from all four channels were sent to the DL model for classification.

In our methodology, we used self-attention-based multi-channel CNN-LSTM for the binary classification task. Figure 6 shows the methodology we used. We will now discuss the proposed model and its components in detail.

#### 2.6.1. Self-Attention Based Multi-Channel Convolutional Neural Network With Long Short Term Memory (MC-CNN-LSTM-Att)

It has been seen that adding convolutional layers to capture local and spatial pattern on top of LSTM layers can be immensely helpful as the CNN-LSTM architecture as a whole involves using CNN layers for feature extraction on input data combined with LSTMs to support sequence prediction. The CNN-LSTM model reads subsequences of the main sequence in as blocks, extracts features from each block, then allows the LSTM to interpret the features extracted from each block. We used a multi-channel approach where we fed the input scalograms from all the four channels altogether to our model. We now discuss the three main components of the model in detail, i.e., CNN, LSTM, and self-attention separately.

##### 2.6.1.1. Convolutional Neural Network

A CNN (Krizhevsky et al., 2012) is a kind of artificial neural network that uses a system much like a multilayer perceptron that has been designed for reduced processing requirements. A classical CNN may contain mainly a convolution layer, pooling layer, fully connected layer, and normalization layer. Recently, CNNs have shown state-of-the-art results on challenging activity recognition tasks with very little or no data feature engineering, by instead using feature learning on raw data; for this reason, we used CNN in the present analysis. The full CNN framework and formula derivation can be seen in the literature (Albawi et al., 2017). In a CNN, the convolutional product between the image and the filter is carried out and a 2D matrix is obtained where each element is the sum of the elementwise multiplication of the filter and of the given input in matrix form. Often a bias is also added to the the result. For simplicity, we only provide the formula for convolutional layer, which works as a filter and is then activated by a non-linear activation function, as follows:


(6) $$\begin{array}{l} {\text{g}}_{x,y}=\text{f}\left(\Sigma_{i=1}^{n_{H}}\Sigma_{j=1}^{n_{W}}\Sigma_{k=1}^{n_{C}}{\text{K}}_{i,j,k}{\text{I}}_{x+i-1,y+j-1,k}+\text{b}\right) \end{array}$$

where *g*_*x, y*_ is the corresponding activation, *K*_*i, j, k*_ denotes the ixjxk weight matrix of convolution kernel, *I*_*x*+*i*−1, *y*+*j*−1, *k*_ indicates the activation of the upper neurons connected to the neuron (x, y), (*n*_*H*_, *n*_*W*_, *n*_*C*_) is the dimension of the input (image), *n*_*H*_ is the height (64 in our case), *n*_*W*_ is the width (64 in our case), and *n*_*C*_ is the number of channels (4 in our case b is the bias value, and f is a non-linear function.

##### 2.6.1.2. Long Short Term Memory

Long short term memory (Hochreiter and Schmidhuber., 1997) network models are a type of recurrent neural network that are able to learn and remember over long sequences of input. They are intended for use with data that has long sequences and are a good fit for time-series problem. The model learns to extract features from sequences of observations (CNN-derived features our case) and how to map the internal features to different conditions (sabotage/no-sabotage). The benefit of using LSTMs for time-series sequence classification is that, since LSTM models able to learn directly from the raw time-series data, they do not require domain experts to manually engineer input features. The model can learn an internal representation of the time series and can achieve comparable performance to models fit on a dataset with engineered features.

A vanilla LSTM (Greff et al., 2015) block has three gates (input, forget, and output), block input, a single cell, an output activation function, and peephole connections. The output of the block is recurrently connected back to the block input and all of the gates. The vector formulas for LSTM layer forward pass are given in Greff et al. (2015). The equations for the LSTM are below:


(7) $$\begin{array}{l} {\text{i}}_{t}=\sigma \left(x^{t}W_{xi}+h_{t-1}W_{hi}+c_{t-1}Wci+b_{i}\right)\;\text{input gate} \end{array}$$

(8) $$\begin{array}{l} {\text{f}}_{t}=\sigma \left(x^{t}W_{xf}+h_{t-1}W_{hf}+c_{t-1}Wcf+b_{f}\right)\;\text{forget gate} \end{array}$$

(9) $$\begin{array}{l} {\text{o}}_{t}=\sigma \left(x^{t}W_{xo}+h_{t-1}W_{ho}+c_{t-1}Wco+b_{o}\right)\;\text{output gate} \end{array}$$

(10) $$\begin{array}{l} {\text{c}}_{t}={\text{f}}_{t}*{\text{c}}_{t-1}+{\text{i}}_{t}*\text{tanh}\left(x^{t}W_{xc}+h_{t-1}Whc+b_{c}\right)\;\text{cell state} \end{array}$$

(11) $$\begin{array}{l} {\text{h}}_{t}={\text{o}}_{t}*\text{tanh}\left({\text{c}}_{t}\right)\;\text{block output} \end{array}$$

In the above equations, σ is the sigmoid activation function, *x*^*t*^ is the input to the LSTM block, *i*_*t*_, *f*_*t*_, *o*_*t*_, *c*_*t*_, and *h*_*t*_ are the input gate, the forget gate, the output gate, the cell state, and the output of the LSTM block, respectively, at the current time step t. *W*_*xi*_,*W*_*xf*_, and *W*_*xo*_ are the weights between the input layer and the input gate, the forget gate, and the output gate, respectively. *W*_*hf*_, *W*_*hi*_, and *W*_*ho*_ are the weights between the hidden recurrent layer and the forget gate, the input gate, and the output gate of the memory block, respectively. *W*_*ci*_, *W*_*cf*_, and *W*_*co*_ are the weights between the cell state and the input gate, the forget gate, and the output gate, respectively, and finally, *b*_*i*_, *b*_*f*_, and *b*_*o*_ are the additive biases of the input gate, the forget gate, and the output gate, respectively.

##### 2.6.1.3. Self-Attention

The self-attention (Vaswani et al., 2017; Singh et al., 2021) mechanism allows the inputs to interact with each other and find out who they should pay more attention to or which features are more important. The attention layer aims to learn the important time points from the sensor time-series data that aid in determining the state label. After leveraging both local contextual features and temporal dynamics by fusing CNN layer and LSTM units from the input time series, we used self-attention layer to learn weight coefficients that were the importance of each feature in input data samples. The attention score, s, for a sample is then given by:


(12) $$\begin{array}{l} {\text{a}}_{s}=\text{softmax}(V_{att}tanh\left(U_{att}h_{t}^{\prime }\right) \end{array}$$

(13) $$\begin{array}{l} \text{s}={\text{a}}_{s}h_{t}^{\prime } \end{array}$$

In the above equations, *U*_*att*_ ∈ *R* ^*D* × *E*^ and *V*_*att*_ ∈ *R* ^*F* × *D*^ are weight matrices forming the attention module, F represents the attention length, D represents the length of the output, represents the number of hidden units in the previous layer (LSTM in our case), and $h_{t}^{\prime }$ is the encoded input from LSTM. Equation 12 finds the softmax of the compatibility (similarity) of the input, and Equation 13 gives the combination of the transformed input.

##### 2.6.1.4. Architecture Used for MC-CNN-LSTM-Att

In our proposed model, the input took the form of 64 × 64 scalogram-like matrix from each of the four channels. The overall shape of input was 64 × 64 × 4, where the last dimension denotes the number of channels. This input was fed to a Conv2D layer (Conv1D in case of raw time-series input) with 32(5 × 5) filters followed by a maxpool layer of 2 × 2, and the output of this layer was fed to another Conv2D layer (Conv1D in case of raw time-series input) with 32(3x3) filters followed by a maxpool again. A dropout layer was then applied to avoid overfitting. The output of the dropout layer and then flattened. The flattened data were then sent to two LSTM layers, after which self-attention was applied to the encoded data from LSTM. The last layer was a dense/fully connected (FC) layer. The output of FC was passed through softmax layer to get the prediction, i.e., sabotage or no-sabotage. Batch normalization was applied in training to normalize the outputs of the layer. The CNN acted as a frontend to the CNN-LSTM model. The CNN model could handle a single image input. It converted it from input pixels into a vector representation. This was carried out with the help of Flatten layer that transformed the image input into a 1D vector representation. In our case of sequential image data, this operation was repeated across multiple images in order to allow the LSTM to build up internal state and update weights across a sequence of the internal vector representations of input images. Each scalogram in our case is treated as a single spatial image input, and the temporal aspect is taken care of by successive overlapping scalogram inputs.

Although CNN and LSTM effectively capture spatio-temporal information, there is a need to target specific information from the embeddings generated by the combination of CNN and LSTM and bring them together since multiple components can together form relevant semantics for decoding the activity being performed. This has been done using the self-attention mechanism that forms a 2D matrix to represent the embedding of the input such that each row of the matrix caters to a different part of the time-series. Along with CNN and LSTM, we show that self-attention leads to a statistically significant results.

## 3. Evaluation Metrics

The accuracy, precision, recall, and F1 score of the model were used as evaluation metrics. The accuracy indicates the samples that were correctly classified from all the samples.


(14) $$\begin{array}{l} \text{Accuracy}\;=\;\frac{\text{TP+TN}}{\text{TP+TN+FP+FN}} \end{array}$$

where TP = TN = true negatives, FP = false positives, and FN = false negatives. A TP is an outcome where the model correctly predicts the positive class. Similarly, a TN is an outcome where the model correctly predicts the negative class. A FP is an outcome where the model incorrectly predicts the positive class. FN is an outcome where the model incorrectly predicts the negative class.

Precision expresses the proportion of the data points our model says was relevant actually were relevant.


(15) $$\begin{array}{l} \text{Precision}\;=\;\frac{\text{TP}}{\text{TP+FP}} \end{array}$$

Recall is the ability of the model to find all the data points of interest in a dataset. It is also called sensitivity or true positive rate (TPR).


(16) $$\begin{array}{l} \text{Recall}\;=\;\frac{\text{TP}}{\text{TP+FN}} \end{array}$$

The F1 score is the harmonic mean of precision and recall taking both metrics into account in the following equation.


(17) $$\begin{array}{l} \text{F1 Score}\;=\;2\times \frac{\text{Precision x Recall}}{\text{Precision + Recall}} \end{array}$$

## 4. Experimental Results

**Figure 7:**
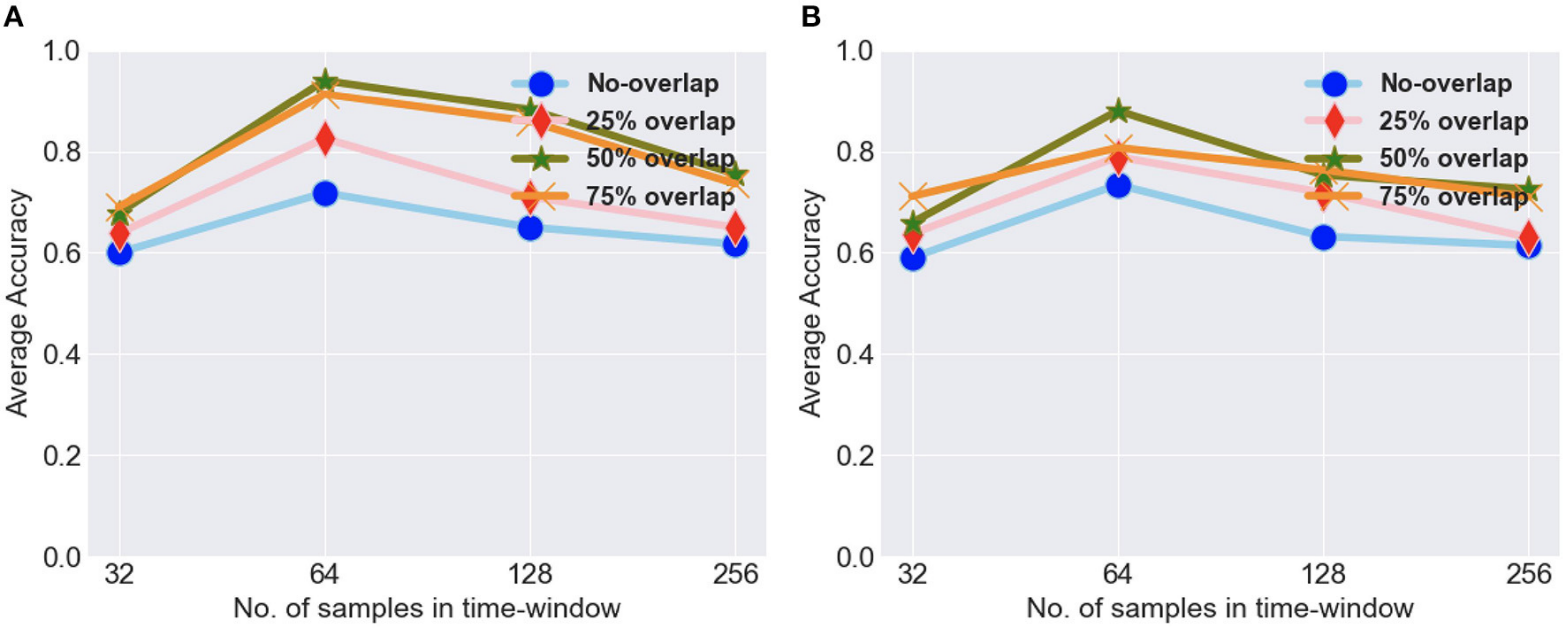
**(A)** The average intra-subject accuracy for different timewindows and overlap values for scalogram data. **(B)** The average intra-subject accuracy for different timewindows and overlap values for raw data.

To evaluate the proposed approach, we performed several experiments, which mainly consisted of classification and statistical analysis. In order to analyze the benefit of time-frequency analysis on classification result, we applied our proposed model both on data with time-frequency analysis (raw data scalograms) and on raw time-series data without time-frequency analysis. We discuss the classification results on both types of data in this section, and then, we perform the statistical analysis of the results. As previously mentioned, we divided the data into overlapping windows of 64 data points with different values of overlap, and in Figure 7 we show the results for both types of dataset with different overlap for different window sizes. We experimented with different window sizes (32, 64, 128, and 256) and different overlap values (no-overlap, 25, 50, and 75%) for the both the types of data, i.e., with and without time-frequency analysis. We got the best average accuracy for a window of 64 with 50% overlap, so we went ahead with this time-window for further experimentation. We divided the data into training data and test data. The train and test splits were in the ratio 70 and 30%, respectively. The divide into train and test for intra-subject was done in a manner that for each of the task with 80 sets of trials, 24 (6 sets from each of the 4 conditions) sets of trials were kept of test and 56 set of trials were kept for training purpose. The data were trained using five-fold cross validation on the training data with 20 epochs for each fold, and after each epoch, the model was cross-validated on the validation data. The metrics in the results are reported on the test data. We performed intra-subject classification on individual participant data and inter-subject classification on the data from all the participants. In case of inter-subject classification, we trained the model by combining data from all participants except one and used these data for testing. For the training of model, we used 12-fold leave-one-subject-out cross validation.

### 4.1. Classification Results on Raw Data

In our experiments, we first used the raw data from the four sensors of Muse to train our DL model. Table 1 shows the performance of our model on raw EEG data, i.e., data without scalogram representation. The highest intra-subject accuracy obtained with this data set was 94.43% (Subject 5), and the minimum intra-subject accuracy was 85.56% (Subject 4). The average intra-subject accuracy was 88.92%, showing that the model performed relatively well for all the subjects. The inter-subject accuracy obtained was 82.50%. The inter-subject classification was carried out to see the generalized performance of the model.

**Table 1:** Performance of the model on raw data.

**Subject**	**Accuracy**	**Precision**	**Recall**	**F1-Score**
Subject 1	92.05	0.934	0.909	0.919
Subject 2	94.05	0.921	0.941	0.931
Subject 3	88.12	0.914	0.791	0.848
Subject 4	85.56	0.923	0.779	0.853
Subject 5	94.43	0.961	0.923	0.939
Subject 6	91.36	0.924	0.908	0.915
Subject 7	91.66	0.952	0.871	0.909
Subject 8	87.83	0.879	0.866	0.872
Subject 9	88.78	0.897	0.882	0.893
Subject 10	80.76	0.729	1.0	0.843
Subject 11	86.67	0.872	0.859	0.865
Subject 12	85.85	0.808	0.989	0.889
Inter-subject	82.50 (± (3.06))	0.862 (± (0.026))	0.785 (± (0.044))	0.821 (± (0.031))
Average Intra-subject	88.92 (± (3.97))	0.893 (± (0.067))	0.896 (± (0.069))	0.889 (± (0.033))

### 4.2. Classification Results on Scalogram of Raw Data

The DL model was applied on the scalogram representation of the raw data from the four EEG channels. Compared to using raw EEG data, accuracy was higher when scalogram data were used (Table 2). We got the highest intra-subject accuracy of 98.71% (Subject 2) and the lowest intra-subject accuracy of 89.10% (Subject 4). The average accuracy of all subjects was 93.83% that shows that the performance of the model improved the average accuracy for all subjects. The inter-subject accuracy also improved and became 86.15%. The results were relatively very good using scalograms and the performance of the model improved on the scalograms.

**Table 2:** Performance of the model on scalograms of raw data.

**Subject**	**Accuracy**	**Precision**	**Recall**	**F1-Score**
Subject 1	96.80	0.975	0.961	0.968
Subject 2	98.71	1.0	0.975	0.987
Subject 3	93.23	0.959	0.870	0.912
Subject 4	89.10	0.967	0.848	0.903
Subject 5	96.01	0.977	0.942	0.959
Subject 6	93.57	0.949	0.925	0.936
Subject 7	93.15	0.971	0.884	0.925
Subject 8	92.94	0.915	0.935	0.925
Subject 9	93.89	0.949	0.933	0.941
Subject 10	95.27	0.915	0.980	0.946
Subject 11	90.23	0.899	0.902	0.901
Subject 12	93.10	0.895	0.992	0.9414
Inter-subject	86.15 (± (2.03))	0.904 (± (0.043))	0.819 (± (0.026))	0.859 (± (0.014))
Average Intra-subject	93.83 (± (2.64))	0.943 (± (0.034))	0.928 (± (0.045))	0.936 (± (0.027))

### 4.3. Statistical Significance of the Classification Results

**Figure 8:**
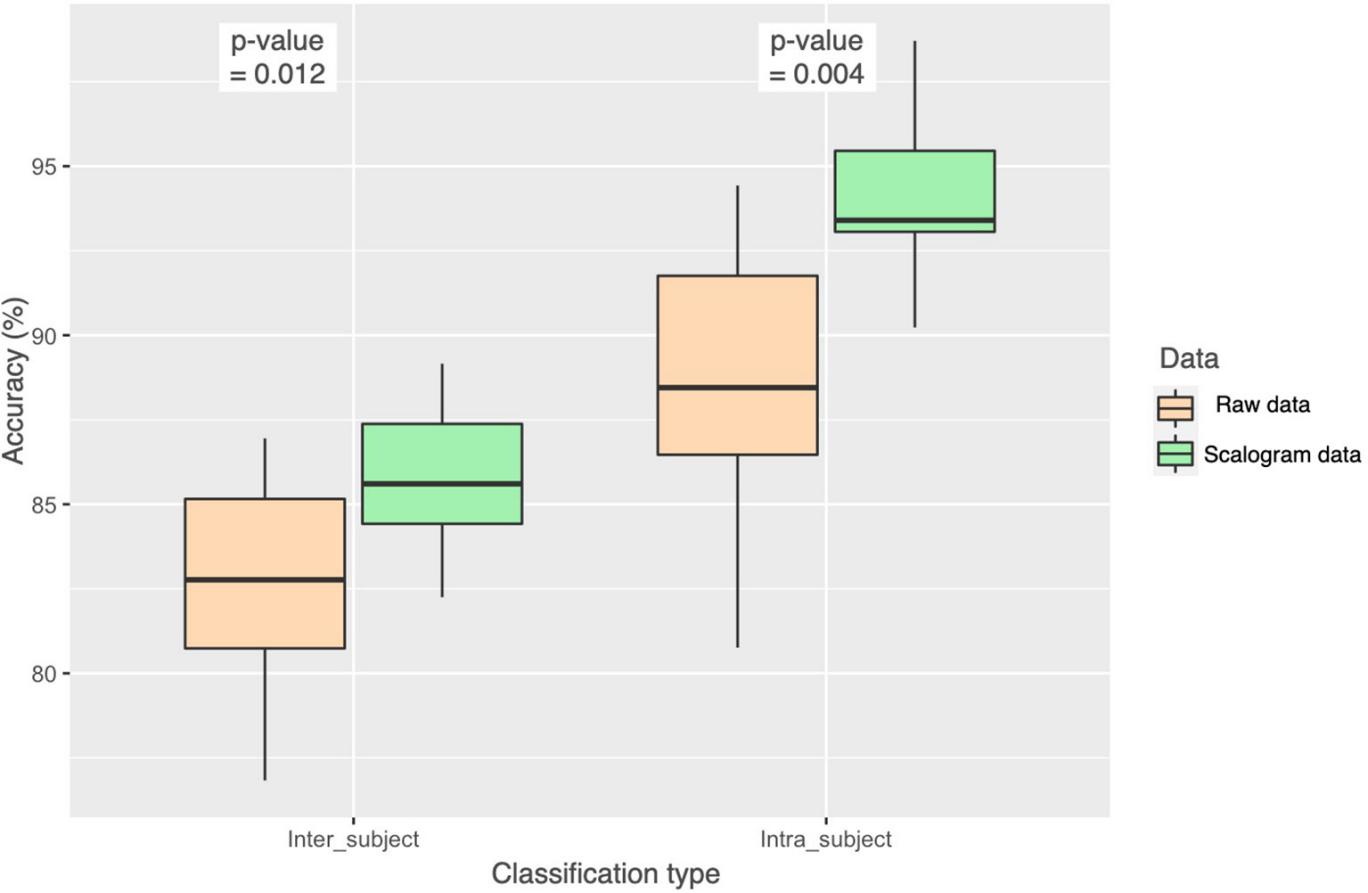
Statistical analysis (ANOVA) of the accuracy achieved from the two approaches, using raw data and using scalogram of raw data, with the *p*-value for both inter-subject and intra-subject classification.

In Figure 8, we show the results of one-way ANOVA (for *p* < 0.05) with the null hypothesis for the test being that the two means are equal. We see that the results were significantly better for scalogram representation of data for both inter-subject and intra-subject cases with *p* = 0.012 and *p* = 0.004, respectively. Our proposed model is scalograms with self-attention based multi-channel CNN-LSTM.

## 5. Discussion and Future Work

The primary aim of this study was to determine whether intentionally performing poorly on a specific CMI assessment task could be detected by analyzing EEG data collected during the assessment. Using a multi-channel DL approach, we found that analysis of EEG spectral data enabled us to differentiate intentionally poor task performance from maximal effort performance with 98.71% accuracy. The present results revealed that the time-frequency representation as the input to the model led to better results than the use of raw data. For future work, we recommend applying different feature extraction techniques on the data or using topographical maps as input to the model to further improve classification accuracy. We could also use transfer learning in future, where we train the larger network on group-level data and then adjust just the final layers with some additional small training data to customize for an individual subject.

The visuomotor skill assessment used in this study has shown promise in as an objective tool to determine whether a concussion has been sustained and as whether full recovery has occurred. Used as a baseline test, individuals at known risk of concussion would complete this assessment when they are known to be concussion-free and then again once an injury is suspected. However, this protocol is only useful if both tests reflect the best effort of an individual, and the threat of litigation or a desire to resume or avoid certain activities prevents some individuals from doing so, under performing on a pre-injury test to enable earlier return to activity or under performing on a post-injury test to delay a diagnosis of recovery. The combination of consumer-grade neural activity collection and DL-based spectral analysis used here provides a potentially important tool for clinicians to use with skill assessment.

A strength of this analysis was that it used technology consumer-grade EEG technology (MUSE 2™ EEG headband), along with a tablet-based task to assess cognitive-motor integration. The technology is portable and affordable; as a result, this is a protocol that can be easily translated to clinical patient care settings. With the advancement in wearable computing, more portable EEG devices are available and future studies replicating these findings with such devices would be useful to further expand the utility of this form of performance monitoring.

The present experiment shares similarities with literature examining the use of neuroimaging techniques as lie detectors. In this study, as in many lie-detection studies, the experimental paradigm had participants performing with low effort to follow instructions rather than with intent to deceive. In a clinical concussion assessment situation, willfully underperforming would be done deceptively, which could alter the cortical activity associated with the task performance. Similarly, in this study, the emotional context did not differ between when the participant was attempting to perform well as compared to when they were not. In real life, concussion testing is personally relevant and highly emotional for the individual being tested, as it is used to make decisions about whether athletes can return to play or whether litigants receive compensation for their injuries. Heightened emotional value of one condition may also affect the neuroimaging results (Greely and Illes, 2007; Farah et al., 2014). However, the present experiment also differs from the lie detection literature. The present experiment shows that there are detectable differences in cortical excitability between conditions when participants perform to their fullest ability on a specific CMI task and when they do not. These two conditions represent different levels of effort, arousal, and attention, but the goal of the was to not to identify which neurocognitive mechanism was most responsible for differences between the conditions. Rather, the goal of this study was to determine whether underperforming on purpose could be detected through machine learning analysis of EEG data, as this has important implications for assessment paradigms in sports medicine and health-care.

We acknowledge that while similar eye-hand coordination was required to successfully complete both experimental conditions, it could be that subtle motor performance differences may have also affected the EEG signals associated with movement control, in addition to the cognitive sabotage aspect to the study. However, the features of the EEG signal that distinguished true- from under-performance conditions were time-frequency scalograms taken from frontal and temporal EEG electrodes recorded for the entire task. Within the spatial sensitivity limits of EEG, we suggest that distinctions quantified by the classifier were less likely related to small changes in the sensorimotor movement coordination networks, which are localized over the parietal lobes, and more likely related to inhibition, behavioral control, and task monitoring functions that are localized to frontal and temporal/limbic regions. Thus, we believe this approach would be useful for classifying effort-related behavior using frontally and temporally generated signals over a wide range of motor tasks. To confirm this assertion, future work will explore true vs. sabotage performance as a function of performance to more clearly distinguish motor aspects from cognitive effort aspects of the task.

This proof-of-concept study showed that a portable EEG headband, combined with a multi-channel CNN-LSTM model, can be used on EEG data to detect sabotage at an average approximately 93% of the time during the performance of a cognitive-motor integration task. Expanding our data collection to a larger sample size will allow for further refinement of the model, as well as the development of a user interface and standard operating procedure to translate this work into clinical practice. Future work can validate the paradigm, establishing sensitivity and specificity in larger groups of people and clinical populations, including those with cognitive impairments or mental illnesses (e.g., depression) and across groups with different individual traits (e.g., extraversion or introversion) (Farah et al., 2014). For example, in applying the present approach to baseline testing performance in athletes, it will be important to validate these findings in groups affected by concussion to distinguish sabotage differences from injury-related differences. This validation would be required before widespread use. In addition, validation studies need to examine the effect of emotion and context, as discussed above, to determine whether willfully underperforming for personal gain changes the EEG profiles associated with the task conditions. The history of lie detection holds lessons about the potential harms of over-reliance on a method to detect deception before the accuracy of that method was established (Greely and Illes, 2007; Farah et al., 2014). Finally, privacy issues must also be considered. Greely and Illes (2007) point out that society “rightly places limits on the collection and use of personal information,” and the benefit of collecting neurophysiological data during a concussion assessment must be considered against the privacy cost to the patient. Future work will also focus on applying this sabotage detection process to a range of brain-computer interfaces for real-time, *in-situ* deceit detection. Such an application would increase the utility of both injury diagnosis and recover assessment using behavioral performance measures.

## Data Availability Statement

The raw data supporting the conclusions of this article will be made available by the corresponding author, without undue reservation.

## Ethics Statement

The studies involving human participants were reviewed and approved by York University's Human Participants Research Committee. The patients/participants provided their written informed consent to participate in this study.

## Author Contributions

MC, LS, and MA did the data collection. MC applied models on the data and wrote the manuscript. SM suggested the methodology and helped in writing the manuscript. MA helped with the neuroscience aspect of the paper and edited the manuscript. ML suggested improvements in the methodology and edited the manuscript. LS suggested improvements and edited the manuscript. All authors contributed to the article and approved the submitted version.

## Conflict of Interest

The authors declare that the research was conducted in the absence of any commercial or financial relationships that could be construed as a potential conflict of interest.

## Publisher's Note

All claims expressed in this article are solely those of the authors and do not necessarily represent those of their affiliated organizations, or those of the publisher, the editors and the reviewers. Any product that may be evaluated in this article, or claim that may be made by its manufacturer, is not guaranteed or endorsed by the publisher.
