# Supplementary material for: Sabotage Detection Using DL Models on EEG Data From a Cognitive-Motor Integration Task
Source: Front Hum Neurosci. 2021 Oct 8;15:662875. doi: 10.3389/fnhum.2021.662875 (PMC8531592; doi:10.3389/fnhum.2021.662875)
Supplement: Supplementary file 1 [file Data_Sheet_1.PDF]

Supplementary Material

1 SUPPLEMENTARY FIGURES

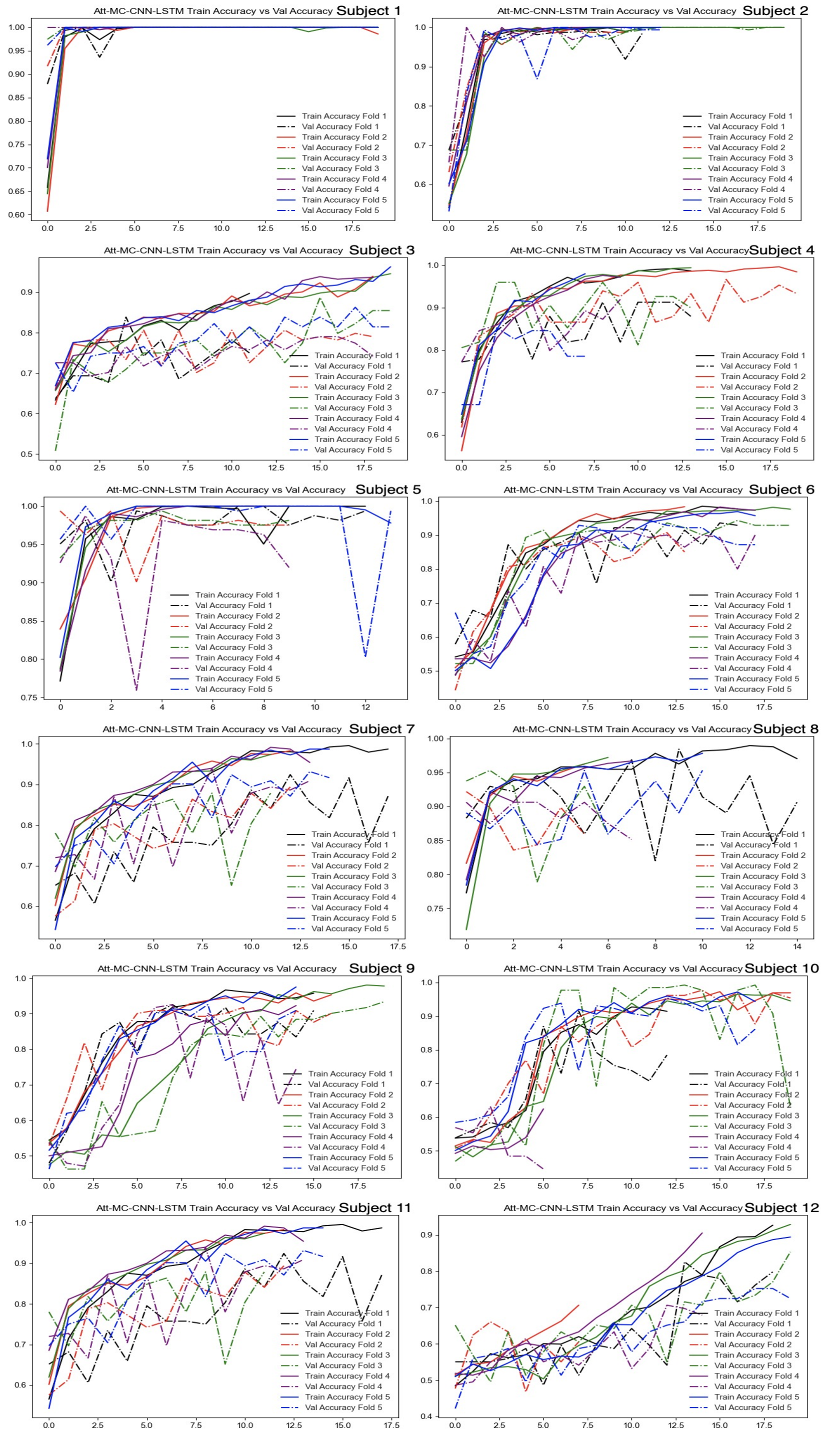

Figure S1. Training summary of all subjects

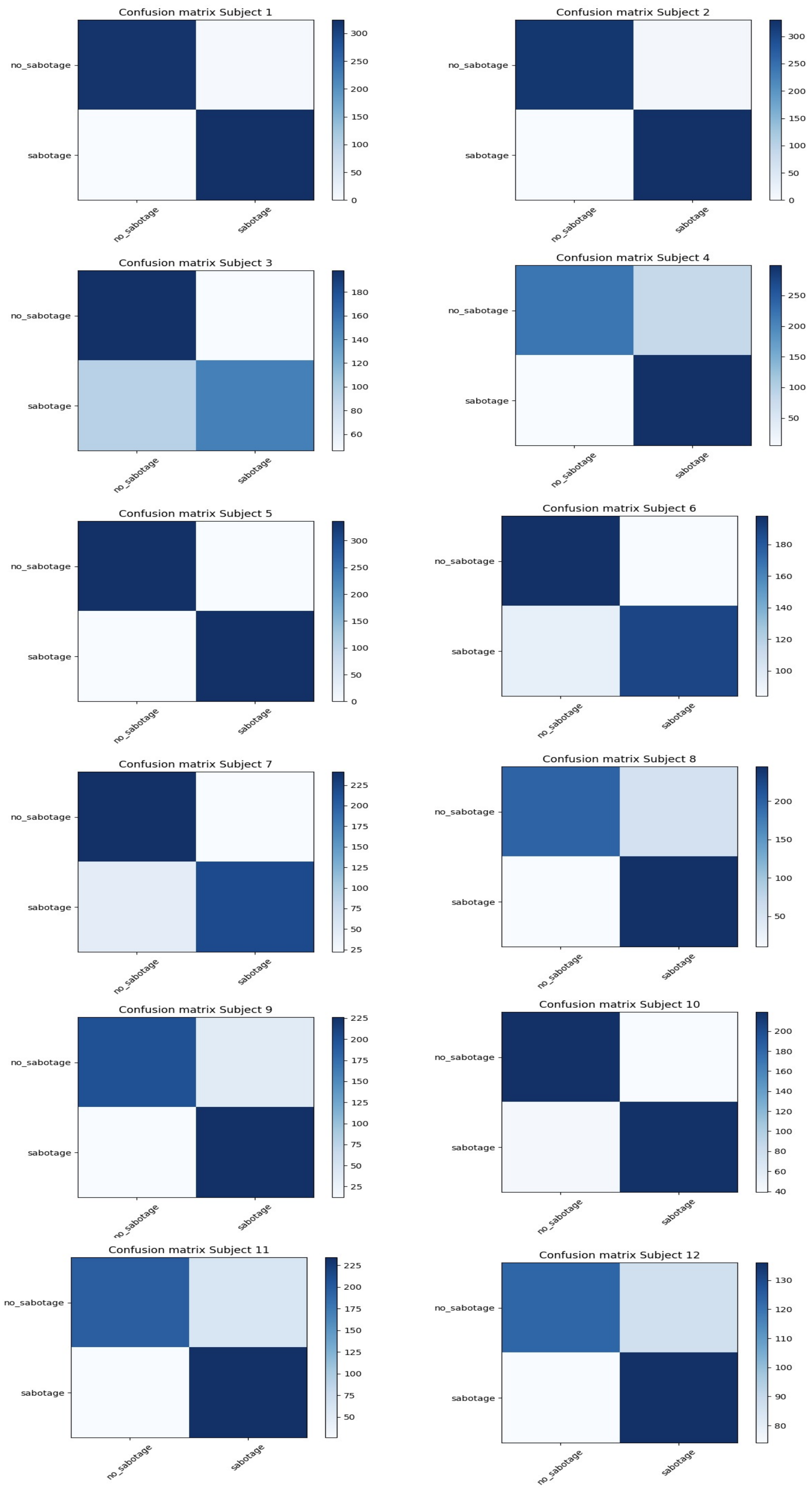

Figure S2. Confusion matrix of the test data of all subjects
